# Naturalistic music and dance: Cortical phase synchrony in musicians and dancers

**DOI:** 10.1371/journal.pone.0196065

**Published:** 2018-04-19

**Authors:** Hanna Poikonen, Petri Toiviainen, Mari Tervaniemi

**Affiliations:** 1 Cognitive Brain Research Unit, Department of Psychology and Logopedics, Faculty of Medicine, University of Helsinki, Helsinki, Finland; 2 Department of Music, Art and Culture Studies, University of Jyväskylä, Jyväskylä, Finland; 3 Cicero Learning, Faculty of Educational Sciences, University of Helsinki, Helsinki, Finland; University of Western Ontario, CANADA

## Abstract

Expertise in music has been investigated for decades and the results have been applied not only in composition, performance and music education, but also in understanding brain plasticity in a larger context. Several studies have revealed a strong connection between auditory and motor processes and listening to and performing music, and music imagination. Recently, as a logical next step in music and movement, the cognitive and affective neurosciences have been directed towards expertise in dance. To understand the versatile and overlapping processes during artistic stimuli, such as music and dance, it is necessary to study them with continuous naturalistic stimuli. Thus, we used long excerpts from the contemporary dance piece *Carmen* presented with and without music to professional dancers, musicians, and laymen in an EEG laboratory. We were interested in the cortical phase synchrony within each participant group over several frequency bands during uni- and multimodal processing. Dancers had strengthened theta and gamma synchrony during music relative to silence and silent dance, whereas the presence of music decreased systematically the alpha and beta synchrony in musicians. Laymen were the only group of participants with significant results related to dance. Future studies are required to understand whether these results are related to some other factor (such as familiarity to the stimuli), or if our results reveal a new point of view to dance observation and expertise.

## Introduction

Neuroscientists have learned a great deal about brain plasticity by studying the brain functions of musical expertise, not only during listening to and performing music but also during unrelated tasks and at rest [1]. Dedication for years to master an instrument has been shown to shape brain structure and sensory, motor, cognitive and affective processes in the brain [2–4]. Recently, the collaborative aspect in music making has become a focus of neuroscience to elucidate the elements of social interaction [5,6]. Auditory-motor interaction has received attention in neurosciences of music [7]. In dance, both collaboration and rhythmic auditory-motor interaction are crucial, which may explain the emerging interest of neuroscientists towards dance.

Neuroscience of dance is a young but fast-growing field. Expertise in dance has been shown to modify the *brain functions* vastly, especially in the premotor regions, [8] and, in parallel, to modulate interpersonal entrainment in movement [9,10]. Dance expertise requires a versatile set of complex skills related to multimodal processing, spatial awareness, embodied interaction, movement timing and execution, and mnemonic and emotional processing.

Recently, also the *brain structure* of professional dancers and musicians has been investigated in comparative studies. Structural magnetic resonance imaging (MRI) has shown that, compared with laymen, the gray matter in both groups of experts is thicker on superior temporal regions [11]. In addition, Karpati and colleagues found that the gray matter structure in the superior temporal gyrus correlates with performance on tasks related to dance imitation, rhythm synchronization and melody discrimination. The structure of the white matter seems to be different in dancers, musicians and laymen. Giacosa and colleagues [12] suggested that dancers have increased diffusivity and reduced fibre coherence in the corpus callosum, corticospinal tract and superior longitudinal fasciculus. In similar regions, musicians showed reduced diffusivity and greater coherence of fibres. Further, these diffusivity measures were related to differential performance in dance, rhythm and melody tasks.

While functional MRI (fMRI) studies highlight the importance of premotor areas in professional dancers when watching dance [8], musicians have refined function on regions related to memory and auditory-motor integration when listening to music [7,13]. In our two previous studies, we showed that dancers and musicians process fast changes in continuous music differently [14] and that the synchrony of activation is stronger in dancers than in musicians and laymen when watching an audiovisual dance piece [15].

fMRI reveals the brain regions in which the changes in activation occur. Within each brain region, there are several neural assemblies, the activation of which is associated with changes in synchrony over frequency bands of electroencephalography (EEG). Alpha (8–13 Hz) and beta (13–30 Hz) bands have been associated with motor and rhythmic processes [16–19]. While beta band activity is assumed to reflect control of complex movements, activity in the alpha band might be associated with automated motor control of well-established movements [20]. In addition, movement initiation is easier when beta synchrony is suppressed [21]. It has been shown that the beta suppression occurs automatically in the auditory cortex when listening to a musical beat without any motor task [22].

Theta band (4–8 Hz) and its coupling to gamma band (30–48 Hz), are connected to several cognitive and affective functions [23–25]. In addition, both theta and alpha bands are modified during attentional and multimodal processing [26–28]. Thus, careful frequency band analysis of EEG is necessary to reveal any differences among the function of neural assemblies between dancers and musicians.

We were interested in rhythmical movement with continuous and overlapping flow of newly initiated movements since the changes in cortical synchrony are generally found within a couple of seconds after observing the initiation of movement. We hypothesized that with the observation of nonstop flow of newly initiated movements, e.g. energetic dancing, the synchronous processes would differ the most from the condition in which the movement is minor or absent. The second temporal derivative of position, which indicates movement acceleration, is shown to correlate well with the perceptual quantity of movement [29,30]. In the movement theory of Laban [31], acceleration is related to the movement factor of time, the other three movement factors being space, weight and flow.

In this study, we were interested in determining whether the presence of music and dance evokes systematic changes in theta, alpha, beta and gamma bands and whether the processing of dance and music over the frequency bands would differ in dancers and musicians. First, we expected dancers to have significant changes in theta band during dance performance. We were curious whether these changes in dancers would be evoked by music, dance, or both [14,15]. Second, we anticipated music (but not dance) to evoke changes in cortical synchrony in musicians [22]. To this end, we showed long excerpts from the contemporary dance choreography *Carmen* to professional dancers, professional musicians and laymen in an EEG laboratory. The dance excerpts were modified so that they included either dance and music (“Music On”) or dance alone (“Music Off”). In our dance stimuli, we searched for periods with nearly motionless presence (“Low Acceleration”) and parts of the choreography with energetic dancing (“High Acceleration”) and anticipated suppression in the alpha frequency (via mirror neuron system) in the latter condition relative to the former [19].

## Materials and methods

### Participants

Altogether 20 professional musicians, 20 professional dancers and 20 people without a professional background in either music or dance participated in the experiment. Two participants from each group were discarded from the data analysis for the following reasons. EEG data in the resting state were not recorded for two dancers, one musician and one participant in the control group. In addition, one musician and one participant in the control group had numerous missing or noisy EEG channels and thus, were excluded.

The final group of musicians comprised 13 females and 5 males, the group of dancers 13 females and 5 males, and the control group 12 females and 6 males. The background of the participants was screened by a questionnaire of music and dance at both professional and every-day levels. The professional background of musicians varied from singing to playing various instruments, such as piano, violin or saxophone. The professional background of dancers was versatile, from ballet and contemporary dance to street dance. Several musicians reported expertise in more than one instrument and several dancers in more than one dance style.

The age of musicians ranged from 21 to 31 years (mean 25.6 years), the age of dancers from 23 to 40 years (mean 29.2 years), and the age of laymen from 20 to 37 years (mean 25.0 years). Two participants in each of the groups were left-handed. No participants reported hearing loss or history of neurological illnesses. The study protocol was conducted in accordance with the Declaration of Helsinki and approved by the University of Helsinki Review Board in Humanities and Social and Behavioral Sciences. When the protocol was approved by the University of Helsinki Review Board in Humanities and Social and Behavioral Sciences, it was documented that the researchers will not share the data outside the research team. Thus, for ethical reasons, we are unable to make original data publicly available.

### Stimuli

Audiovisual excerpts of *Carmen* composed by Bizet-Shchedrin were used as stimuli. The version of the composition used was performed by Moscow Virtuosi Chamber Orchestra and published by Melodiya, Moscow 1987. Many participants reported being familiar with the composition. The dance choreography of *Carmen* was based on contemporary dance choreographed by Mats Ek. However, the female contemporary dancer who performed the dance excerpts for our research purposes had artistic freedom to create solo versions to suit her own expression. Thus, the dance choreography was not familiar to any of the participants. The dancer’s performance was captured with Motion Capture (Qualisys, Gothenburg, Sweden).

At the beginning of the experiment, a 90-seconds resting EEG was collected with the participant’s eyes open and a 90-second resting EEG with eyes closed. The resting-state EEG was collected to compare resting EEG between the groups, reported in [15]. This is a crucial control condition if significant changes occur between groups during tasks. Based on the resting EEG, we know whether the group changes during a task emerge due to the task itself or due to different brain functions between groups in general. These general changes could be assumed to be found already in the resting EEG.

The total length of the stimulus was approximately 15 minutes, which was divided into 20 trials, the duration of each trial being between 15 and 63 seconds (mean 44.5 seconds). Music without visual stimulus (Music On; Dance Off), silent dance (Music Off; Low Acceleration, High Acceleration referring to the motion captured data calculated by MoCap Toolbox and described in detail below), and dance and music as an audiovisual entity (Music On; Low Acceleration, High Acceleration) were presented to the participants. The 60-second period from the resting-state EEG with eyes open was used as the Dance Off, Music Off condition. Music, silent dance and audiovisual dance were presented in a random order. During the presentation of music only the participants were advised to listen to the music with their eyes open, although there was no visual stimulus on the screen. The excerpts were chosen from the composition based on their musical versatility, strong emotional content and variety in movement qualities from subtle gestures to vast energetic dancing. The emotional content interpreted by both music and movement varied significantly, some excerpts transmitting a joyful atmosphere, others anger or devastating sadness.

### Equipment and procedure

The stimuli were presented to the participants with the Presentation 14.0 program. Each set of trials contained 20 excerpts of the same sensory modality/modalities, and these sets were presented in a random order via a monitor and headphones with an intensity of 50 decibels above the individually determined hearing threshold. The distance of the monitor from the participant was 110 cm. The participants were advised to listen to the music with their eyes open and remain still while watching the dance video. The participants did not have a specific task during the experiment, other than being instructed to look and listen to the stimuli. The objective was to have the experimental situation resemble as closely as possible the actual participation in a dance/music performance. The playback of each trial was launched by the researcher. From time to time, between the stimuli, the researcher had a short conversation with the participant via microphone to ensure that the participant felt comfortable during the test procedure. The total length of the study material was 60 minutes. With pauses and conversations based on individual needs of participants, the whole test session lasted about 70–80 minutes.

Data were recorded using BioSemi electrode caps with active 128 EEG channels and 4 external electrodes placed at the tip of the nose, the left and right mastoids, and under the right eye. The offsets of the active electrodes were kept below 25 millivolts at the beginning of the measurement. The data were collected with a sampling rate of 1024 Hz. The beginning and end of each trial were indicated with a trigger in the EEG data.

### Data processing and analysis

We have previously analyzed the data in terms of event-related potentials (ERPs) [14] and group comparisons in phase synchrony [15]. In our current analysis, we were interested in the phase synchrony over the sensory modalities and frequency bands within each group of participants. These three analyses complement each other and can be used as a general reference for future EEG studies in continuous music and dance involving expertise.

### Extraction of movement qualities with MoCap toolbox

We used MoCap Toolbox (version 1.1) to computationally extract the movement qualities. MoCap Toolbox, a set of MATLAB functions designed for the analysis and visualization of Motion Capture data [32], is used for the extraction of different features related to various movement dimensions identified in kinetics and kinematics. The toolbox is mainly used for the analysis of music-related movement and has been applied for capturing different movement qualities defined in movement theory by Laban [31,33].

Acceleration was calculated by MoCap Toolbox for each time point and each selected marker by time-differencing the velocity scalar, which is the norm of the three-dimensional velocity vector, obtained by calculating the first time derivative of the location of the marker. Subsequently, we calculated the absolute value of acceleration for each data point before averaging the values of the markers in the right and left elbow and the right and left knee. Then, we averaged the absolute values of acceleration over the 5-second segments with 50% overlapping in each consecutive segment. Since we were interested in large inter-excerpt variability in movement, we extracted the segments with the largest (10% of the whole Motion Capture data) and smallest (10% of the whole Motion Capture data) absolute values of acceleration to be used as a temporal reference in the synchrony analysis of the EEG data. These segments are referred as High Motion Capture Acceleration (High Acceleration) and Low Motion Capture Acceleration (Low Acceleration), respectively. Perceptually, the epochs of High Acceleration contain large fast movements such as jumps, pirouettes, vast arm and leg movements and moving rapidly in space. Epochs of Low Acceleration contain simple small movements such as turning the head calmly, slow steps or just standing with no or minor body movements. The movements during Low Acceleration are not dance as such, but rather embodied presence and interpretation of emotions relevant to the storyline.

### Preprocessing

The EEG data of all the participants were first preprocessed with EEGLAB (version 12.0.2.5b] [34]. The data were downsampled to 512 Hz and external electrodes of the left and right mastoid were set as a reference. The data were high-pass filtered at 1 Hz and low-pass filtered at 60 Hz. Finite impulse response (FIR) filtering, based on the firls (least square fitting of FIR coefficients) MATLAB function, was used as a filter for all data. The data were then treated with Independent Component Analysis (ICA) decomposition with the runica algorithm of EEGLAB [34] to detect and remove artefacts related to eye movements and blinks. ICA decomposition gives as many spatial signal source components as there are channels in the EEG data. The number of components was 128 in 18 participants. In the remaining 36 participants, some noisy channels from each were removed in preprocessing, and therefore, less than 128 ICA components were decomposed in them. Typically, 1 to 3 ICA components related to the eye artefacts were removed. Noisy EEG data channels of the aforementioned 36 participants were interpolated. After the interpolation, the data were split to the frequency bands of 4–8 Hz (theta), 8–13 Hz (alpha), 13–30 Hz (beta), and 30–48 Hz (gamma) with high-pass and low-pass filtering.

### Synchrony analyses

We calculated the phase synchrony values (PSVs) of the EEG data to the same 5-second segments as were defined before as High Acceleration and Low Acceleration. The PSV was calculated based on the Hilbert transform of the data stream by an electrode pair under comparison and the Shannon entropy of the phase difference distribution. The Hilbert-based method, introduced by Tass and colleagues [35], is widely used in phase synchrony analysis, e.g. [36–38]. A similar method has also been used in EEG data analysis with continuous music stimuli [39].

The synchronization indices are estimated based on the Shannon entropy of the phase difference distribution. Let φ_*i*_*(t)* and φ_*j*_*(t)* denote the instantaneous phases of the signals measures from sensors *i* and *j*. To obtain the synchronization index, we calculate the phase difference distribution of φ_*i*_*(t)–* φ_*j*_*(t)* ϵ [0,2π] using N bins, denoted by (p_k_), k = 1,…,N. The phase synchronization index is obtained by ρ_ij_ = (S–S_max_)/S_max_, where S = – Σ^N^_k = 1_ p_k_ ln p_k_, and S_max_ = ln N. In the present analyses, we used N = 50. The data were analyzed within a time-window of 5 seconds, and then these PSVs for each 5-second trial were averaged so that there was a PSV value for each participant in each condition.

We conducted the synchrony analysis over the 12 electrodes of C29 (Fp1), C16 (Fp2), C23 (FCz), D3 (FC3), C3 (FC4), D11 (FC5), B30 (FC6), D19 (C3), B22 (C4), A3 (CPz), A17 (PO1), and A30 (PO2) (the 128-channel BioSemi EEG gap) so that each electrode was compared pairwise to all other ones, resulting in 66 electrode pairs of comparison. This analysis was conducted separately for each frequency band of theta, alpha, beta, and gamma. Due to noisy or lacking electrodes during the EEG recording, the value for electrode C16 was interpolated over the surrounding electrodes during the preprocessing of the EEG data for two dancers, one musician and one participant in the control group. Similarly, the electrode D11 was interpolated for one musician. All PSVs of the 5-second segments correlated with High Acceleration were averaged over each participant and each stimulus condition. The same procedure was used for the segments correlated with Low Acceleration. Thus, for each condition, each participant received a unitary PSV for each electrode pair for both High Acceleration and Low Acceleration. In addition, PSV was calculated over the 60-second resting EEG data, during which the participant sat quietly, with eyes open, in a dark and silent EEG laboratory. Similarly to the stimulus data, the data of the resting EEG were segmented with 5-second intervals and 50% overlapping of the two consecutive segments separately. For each participant, the resting PSVs calculated for the 5-second segments were averaged as a unitary PSV value for each electrode pair.

The statistical analyses were conducted with MATLAB version R2016a. In the statistical analysis, repeated measures ANOVA (between-subject factor Dance: Dance Off (e.g. rest and unimodal music), Low Acceleration and High Acceleration; within-subject factor Music: Music Off (e.g. rest and unimodal dance of Low Acceleration and High Acceleration) and Music On was conducted separately for each electrode pair (66 electrode pairs), each frequency band (theta, alpha, beta, and gamma) and each group (dancers, musicians and laymen). The main effects for the factor Dance and Music, and the Dance*Music interaction were calculated with the Greenhouse-Geisser (GG) adjustment. The multiple comparisons of Dance and Music were calculated with the critical value of Bonferroni. The comparison of 66 electrode pairs increased the Type 1 error. Thus, False Discovery Rate (FDR) was calculated for each set of 66 electrode pairs from their pGG values to control the expected proportion of false positives. For FDR correction, we employed a q-value threshold of 0.05. In the Results section, we report only the statistically significant results in which both the pGG and the pFDR are <0.05.

## Results

The significant effects of **dancers** are presented in detail in Table 1. Dancers had a significant main factor of *Music in the theta*, *beta*, *and gamma bands*. No significant main factor of Dance or Dance*Music interactions was found in dancers. In addition, dancers had no significant main factor Music in the alpha band.

**Table 1:** Dancers: Electrode pairs with significant synchronization differences for the main factor of Music (Music Off, Music On) over the frequency bands theta (4–8 Hz), beta (13–30 Hz), and gamma (30–48 Hz). In the table, pGG indicates the p-value with a Greenhouse-Geisser adjustment and pFDR the p-value according to the False Discovery Rate.

4–8 Hz Electrode pair	Condition	F(1,51)	pGG	pFDR	Q	
CPz–FCz	Music	5.7953	0.019726	0.017703	0.017703	Music On > Music Off
CPz–Fp1	Music	6.8383	0.011704	0.015005	0.015005	Music On > Music Off
C4 –PO1	Music	4.5473	0.037806	0.021205	0.021205	Music On > Music Off
FC6 –FC5	Music	6.0492	0.017343	0.017294	0.017294	Music On > Music Off
FC6 –PO2	Music	7.7378	0.007561	0.01357	0.01357	Music On > Music Off
FC4 –FC3	Music	6.9756	0.010941	0.016364	0.015005	Music On > Music Off
FC4 –FC5	Music	11.755	0.001209	0.005424	0.003923	Music On > Music Off
FC4 –Fp1	Music	5.2869	0.025616	0.020898	0.018575	Music On > Music Off
FCz–FC3	Music	14.295	0.000411	0.003689	0.003689	Music On > Music Off
FCz–FC5	Music	11.569	0.001311	0.003923	0.003923	Music On > Music Off
FCz–Fp1	Music	6.3587	0.014847	0.016655	0.016655	Music On > Music Off
FC3 –FC5	Music	4.6205	0.036359	0.021753	0.021205	Music On > Music Off
FC5 –C3	Music	5.1922	0.026907	0.018575	0.018575	Music On > Music Off
FC5 –Fp1	Music	4.8328	0.032487	0.020825	0.020825	Music On > Music Off
FC5 –PO2	Music	10.79	0.001849	0.004148	0.004148	Music On > Music Off
C3 –PO2	Music	5.221	0.026508	0.019824	0.018575	Music On > Music Off
13–30 Hz	Condition	F(1,51)	pGG	pFDR	Q	
FC4 –Fp1	Music	8.98	0.0042	0.031	0.031	Music On > Music Off
30–48 Hz Electrode pair	Condition	F(1,51)	pGG	pFDR	Q	
CPz–C4	Music	8.8067	0.004561	0.002924	0.001822	Music On > Music Off
CPz–FC6	Music	4.4854	0.039081	0.002278	0.001822	Music On > Music Off
CPz–FC4	Music	4.3258	0.042583	0.0021	0.001822	Music On > Music Off
CPz–FCz	Music	4.8121	0.032845	0.003008	0.001822	Music On > Music Off
CPz–C3	Music	5.3465	0.024836	0.003184	0.001822	Music On > Music Off
CPz–PO2	Music	4.5074	0.038623	0.002476	0.001822	Music On > Music Off
C4 –FC6	Music	4.2596	0.044136	0.002021	0.001822	Music On > Music Off
C4 –FC4	Music	4.6491	0.03581	0.002551	0.001822	Music On > Music Off
C4 –FCz	Music	7.0322	0.010641	0.002274	0.001822	Music On > Music Off
FC6 –FCz	Music	5.3081	0.025336	0.002707	0.001822	Music On > Music Off
FC6 –PO2	Music	4.4408	0.040027	0.002138	0.001822	Music On > Music Off
FC4 –FCz	Music	5.5293	0.022602	0.003622	0.001822	Music On > Music Off
FCz–FC3	Music	4.6862	0.035109	0.002814	0.001822	Music On > Music Off
FCz–C3	Music	4.2578	0.044177	0.001888	0.001822	Music On > Music Off
FCz–Fp1	Music	8.1615	0.006178	0.00198	0.001822	Music On > Music Off
Fp1 –Fp2	Music	4.2042	0.04548	0.001822	0.001822	Music Off > Music On

All significant effects of **musicians** are presented in detail in Table 2. Musicians had a significant main factor of *Music in the alpha and beta bands*. There was no significant main factor of Dance or Dance*Music interactions found in musicians. In addition, musicians had no significant main factor of Music in the theta nor gamma band.

**Table 2:** Musicians: Electrode pairs with significant synchronization differences for the main factor of Music (Music Off, Music On) over the frequency bands alpha (8–13 Hz) and beta (13–30 Hz). In the table, pGG indicates the p-value with a Greenhouse-Geisser adjustment and pFDR the p-value according to the False Discovery Rate.

8–13 Hz Electrode pair	Condition	F(1,51)	pGG	pFDR	Q	
FC3 –C3	Music	5.2749	0.025776	0.009381	0.002574	Music Off > Music On
13–30 Hz Electrode pair	Condition	F(1,51)	pGG	pFDR	Q	
CPz–PO1	Music	4.8195	0.032716	0.001239	0.000468	Music Off > Music On
FC4 –FC3	Music	5.5373	0.02251	0.001044	0.000468	Music Off > Music On
FCz–C3	Music	4.3797	0.041365	0.001044	0.000468	Music Off > Music On
FC3 –FC5	Music	4.7606	0.033752	0.001022	0.000468	Music Off > Music On
FC3 –C3	Music	6.9828	0.010902	0.001651	0.000468	Music Off > Music On
FC5 –C3	Music	4.2669	0.043961	0.000951	0.000468	Music Off > Music On
PO1 –PO2	Music	4.9303	0.03086	0.001558	0.000468	Music Off > Music On

All significant effects of **laymen** are presented in detail in Table 3. Laymen had a significant main factor of *Music in the theta and gamma bands* and *Dance in the alpha band*. Dance*Music interaction was significant in laymen *in the theta*, *alpha*, *beta and gamma bands*. Laymen had no significant main factor of Music in the alpha or beta band and no significant main factor of Dance in the theta, beta, or gamma bands.

**Table 3:** Laymen: Electrode pairs with significant synchronization differences for the main factors of Music (Music Off, Music On) and Dance (Dance Off, Low Acceleration, High Acceleration) and the Music*Dance interaction over the frequency bands theta (4–8 Hz), alpha (8–13 Hz), beta (13–30 Hz), and gamma (30–48 Hz). In the table, pGG indicates the p-value with a Greenhouse-Geisser adjustment and pFDR the p-value according to the False Discovery Rate.

4–8 Hz Electrode pair	Condition	F(1,51)	pGG	pFDR	Q	
FC6 –Fp1	Music	14.079	0.00045	0.013499	0.013499	Music On > Music Off
4–8 Hz Electrode pair	Condition	F(2,51)	pGG	pFDR	Q	Multiple comparison (Bonferroni)
C4 –PO1	Dance*Music	4.7453	0.012882	0.011505	0.011505	Music Off: Low Acceleration > Dance Off p = .046981
FC6 –PO1	Dance*Music	9.6489	0.000279	0.000998	0.000998	Music Off: Low Acceleration > Dance Off p = .026826
FC5 –Fp1	Dance*Music	10.42	0.000161	0.001148	0.000998	Music Off: Low Acceleration > Dance Off p = .0026445 Music Off: High Acceleration > Dance Off p = .039634
FC5 –Fp2	Dance*Music	6.2767	0.003656	0.008707	0.006854	Music Off: Low Acceleration > Dance Off p = .018591
8–13 Hz Electrode pair	Condition	F(2,51)	pGG	pFDR	Q	Multiple comparison (Bonferroni)
CPz–FC3	Dance	4.4369	0.01673	0.044196	0.033445	Dance Off > Low Acceleration p = .023613
CPz–FC5	Dance	4.1208	0.021931	0.038624	0.033445	Dance Off > High Acceleration p = .037726
FCz–Fp2	Dance	4.1358	0.02165	0.045754	0.033445	Dance Off > High Acceleration p = .03079
FC3 –Fp2	Dance	4.109	0.022156	0.033445	0.033445	Dance Off > High Acceleration p = .018456
FC5 –Fp2	Dance	5.7201	0.005737	0.030314	0.026586	Dance Off > High Acceleration p = .004355
C3 –Fp1	Dance	5.3861	0.007548	0.026586	0.026586	Dance Off > Low Acceleration p = .044809 Dance Off > High Acceleration p = .0099519
C3 –Fp2	Dance	6.9036	0.002222	0.023474	0.023474	Dance Off > Low Acceleration p = .01751 Dance Off > High Acceleration p = .0031588
8–13 Hz Electrode pair	Condition	F(2,51)	pGG	pFDR	Q	Multiple comparison (Bonferroni)
C4 –PO1	Dance*Music	3.4415	0.039625	0.015475	0.015475	Music On: Dance Off > High Acceleration p = .015847
FC6 –FC4	Dance*Music	7.8114	0.001098	0.00729	0.003676	Music On: Dance Off > High Acceleration p = .011349
FC6 –FCz	Dance*Music	3.5678	0.035462	0.015695	0.01475	Music On: Dance Off > High Acceleration p = .009133
FC6 –FC3	Dance*Music	4.9757	1.06E-02	0.00881	0.007949	Music On: Dance Off > High Acceleration p = .038185
FC6 –PO1	Dance*Music	4.8323	0.011973	0.007949	0.007949	Music On: Dance Off > High Acceleration p = .014495
FC4 –PO1	Dance*Music	5.3597	0.007714	0.007316	0.007316	Music On: Dance Off > High Acceleration p = .024206
FC3 –Fp1	Dance*Music	3.5989	0.034507	0.016364	0.01475	Music On: Dance Off > High Acceleration p = .023361
Fp1 –Fp2	Dance*Music	4.2566	0.019517	0.011779	0.011779	Music On: Dance Off > High Acceleration p = .04331
Fp2 –PO1	Dance*Music	7.8005	0.001107	0.003676	0.003676	Music On: Dance Off > High Acceleration p = .02547
13–30 Hz Electrode pair	Condition	F(2,51)	pGG	pFDR	Q	Multiple comparison (Bonferroni)
C4 –FC5	Dance*Music	5.3899	0.007524	0.033552	0.03277	Music On: Dance Off > High Acceleration p = .027635 Music On: Dance Off > Low Acceleration p = .023326
30–48 Hz Electrode pair	Condition	F(1,51)	pGG	pFDR	Q	
C4 –Fp1	Music	11.421	0.001399	0.029417	0.029417	Music On > Music Off
C3 –Fp2	Music	8.1547	0.006197	0.043443	0.043443	Music On > Music Off
30–48 Hz Electrode pair	Condition	F(2,51)	pGG	pFDR	Q	Multiple comparison (Bonferroni)
C4 –FC3	Dance*Music	6.2303	0.003795	0.001017	0.001017	Music Off: Low Acceleration > Dance Off p = .026043

These results will be further specified below, within each frequency band and for all groups of participants, comparing experimental situations in which music/dance was on/off. When the dance was on, it was divided into segments according to the acceleration of movement: Low Acceleration (nearly still presence or tender movement of an individual body part) and High Acceleration (vast energetic dance movement). Results are considered significant only if pGG < 0.05 and pFDR < 0.05.

### Theta phase synchrony, 4–8 Hz

**Fig 1:**
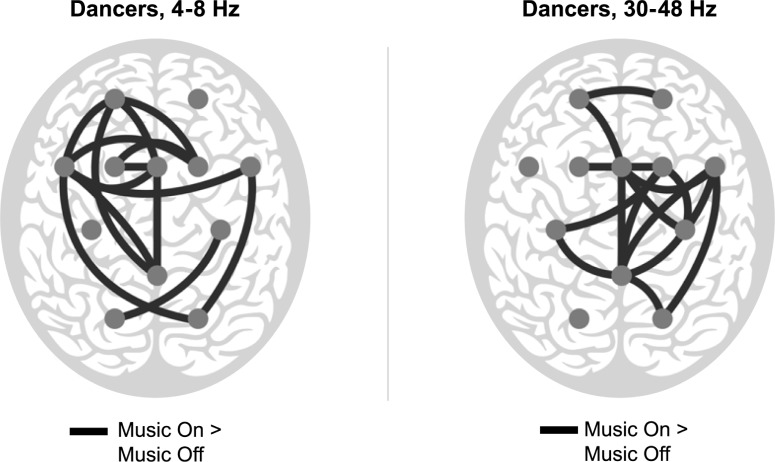
Significant differences for the main factor of Music (Music On, Music Off) for dancers in the theta (4–8 Hz; left) and gamma band (30–48 Hz; right). The black lines connect the electrode pairs over which the synchronization is significantly stronger during Music On than during Music Off. Each gray dot illustrates the location of an EEG electrode on the scalp.

In dancers, in the theta band, the phase synchrony was significantly stronger in Music On than in Music Off over the following electrode pairs: CPz–FCz, CPz–Fp1, C4 –PO1, FC6 –FC5, FC6 –PO2, FC4 –FC3, FC4 –FC5, FC4 –Fp1, FCz–FC3, FCz–FC5, FCz–Fp1, FC3 –FC5, FC5 –C3, FC5 –Fp1, FC5 –PO2, and C3 –PO2 (Fig 1).

**Fig 2:**
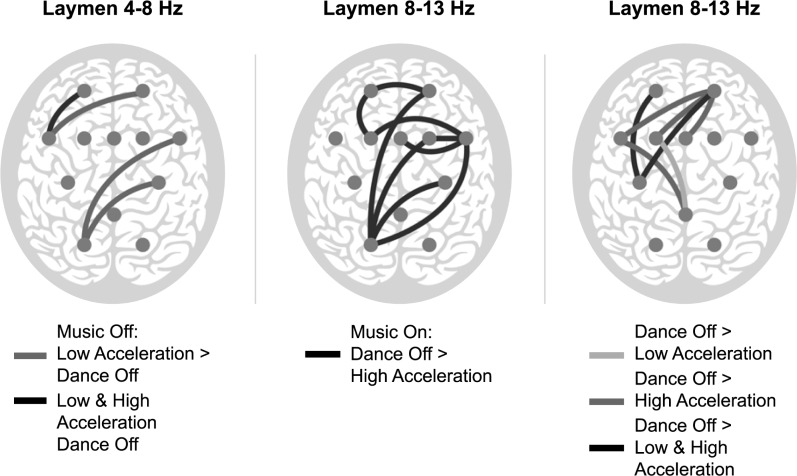
Significant differences for the Dance*Music interaction (Dance Off, Low Acceleration, High Acceleration; Music Off, Music Off) and the main factor of Music (Music On, Music Off) for laymen in the theta (4–8 Hz; on left) and alpha (8–13 Hz; in the middle Dance*Music interaction during Music On; on right main of factor Music) bands. The colour coding of the lines which connect the electrode pairs with significant differences is explained next to each image. Each gray dot illustrates the location of an EEG electrode on the scalp.

In laymen, in the theta band, the phase synchrony was significantly stronger in Music On than in Music Off over the electrode pair FC6 –Fp1. Dance*Music interaction was significant in laymen in the theta band over the electrode pairs FC5 –Fp1, C4 –PO1, FC6 –PO1, and FC5 –Fp2 (Fig 2). In the Music Off condition, over FC5 –Fp1 the synchrony was significantly stronger during both Low and High Acceleration than in Dance Off, and over C4 –PO1, FC6 –PO1 and FC5 –Fp2 synchrony was significantly stronger only during Low Acceleration compared with the Dance Off condition, while in the Music On condition no significant differences were present.

### Alpha phase synchrony, 8–13 Hz

In musicians, in the alpha band, the phase synchrony was significantly stronger in Music Off than in Music On over the electrode pair FC3 –C3.

In the alpha band, laymen had a significant main effect of Dance over several electrode pairs. Dance Off evoked a stronger synchrony than High Acceleration over the electrode pairs CPz–FC5, FCz–Fp2, FC3 –Fp2 and FC5 –Fp2 (Fig 2). Over the electrode pair CPz–FC3 the synchrony during Dance Off was significantly stronger than during Low Acceleration, and over the pairs C3 –Fp1 and C3 –Fp2 Dance Off was significantly stronger than both Low and High Acceleration. In addition, laymen had a significant Dance*Music interaction in the alpha band over the following electrode pairs: C4 –PO1, FC6 –FC4, FC6 –FCz, FC6 –FC3, FC6 –PO1, FC4 –PO1, FC3 –Fp1, Fp1 –Fp2, and Fp2 –PO1 (Fig 2). Over all these pairs, in the Music On condition the synchrony during Dance Off was significantly stronger than during High Acceleration.

### Beta phase synchrony, 13–30 Hz

In dancers, in the beta band, only the electrode pair FC4 –Fp1 produced a significant main factor of Music (Music On > Music Off).

**Fig 3:**
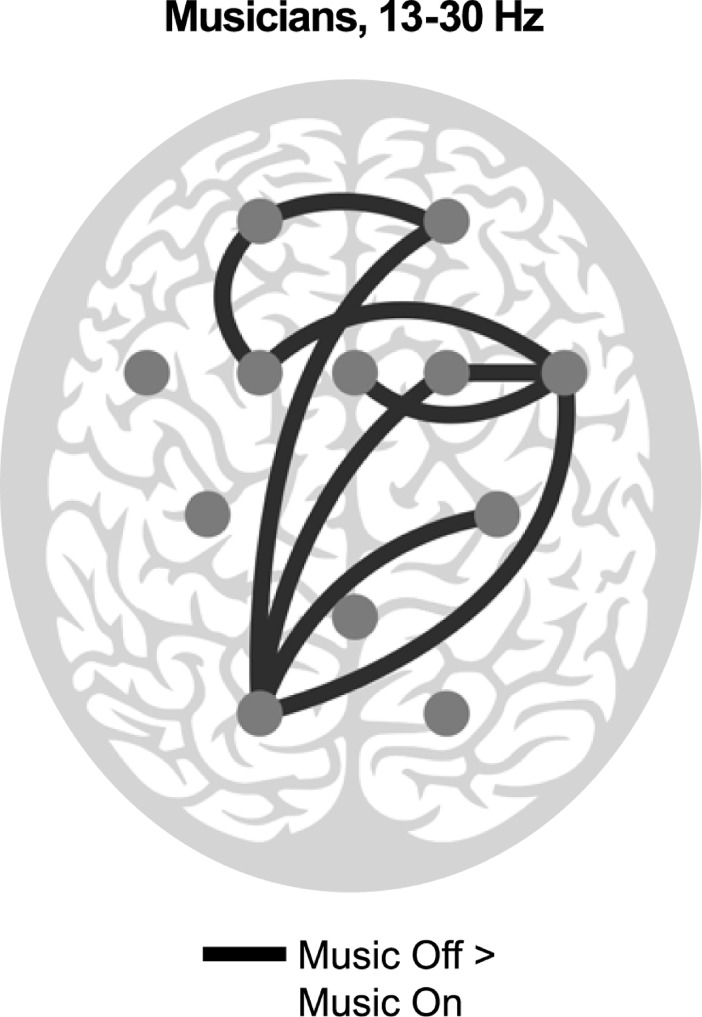
Significant differences for the main factor of Music (Music On, Music Off) for musicians in the beta band (13–30 Hz). The black lines connect the electrode pairs over which the synchronization is significantly stronger during Music On than during Music Off. Each gray dot illustrates the location of an EEG electrode on the scalp.

In musicians, in the beta band, the phase synchrony was significantly stronger in Music Off than in Music On over the following electrode pairs: CPz–PO1, FC4 –FC3, FCz–C3, FC3 –FC5, FC3 –C3, FC5 –C3, and PO1 –PO2 (Fig 3).

In laymen, over the pair C4 –FC5, the Dance*Music interaction was significant in the beta band, in which the synchrony was stronger during Dance Off than during either Low or High Acceleration in the Music On condition.

### Gamma phase synchrony, 30–48 Hz

In dancers, in the gamma band, the synchrony was significantly stronger in Music On than in Music Off over the following electrode pairs: CPz–C4, CPz–FC6, CPz–FC4, CPz–FCz, CPz–C3, CPz–PO2, C4 –FC6, C4 –FC4, C4 –FCz, FC6 –FCz, FC6 –PO2, FC4 –FCz, FCz–FC3, FCz–C3, FCz–Fp1, and Fp1 –Fp2 (Fig 1).

In laymen, in the the gamma band, the phase synchrony was significantly stronger in Music On than in Music Off over the electrode pairs C4 –Fp1 and C3 –Fp2. Dance*Music interaction was significant in laymen on gamma band over the pair C4 –FC3, in which the synchrony was significantly stronger during Low Acceleration than during Dance Off in the Music Off condition.

## Discussion

We investigated EEG phase synchrony over the frequency bands of theta, alpha, beta, and gamma in dancers, musicians, and laymen while they watched a dance piece. The results suggest that cortical processing of music is different in professional dancers than in professional musicians.

Whereas dancers had stronger synchrony during Music On relative to Music Off in the theta and gamma bands, musicians had decreased synchrony during Music On relative to Music Off in the alpha and beta bands. Interestingly, laymen were the only participant group that had significant differences in synchrony related to the dance movement. In silence, particularly Low Acceleration, which refers to ordinary minor movement such as turning the head or gently lifting an arm, increased the synchrony in the theta and gamma bands compared with Dance Off. In the alpha and beta bands, synchrony was significantly decreased over several electrode pairs during High Acceleration of Music On, which refers to vast dance movement synced with music, compared with Dance Off.

### Music effects

To understand the differences in cortical synchrony in dancers and musicians when listening to music, we may ask: What is music for a musician? What is music for a dancer? And, further, do these elements explain the differences in cortical synchrony over the frequency bands?

When listening to music, a musician may be mentally playing the tones with his instrument, especially if the musical piece is familiar. In our study, several test subjects in each group reported the composition a familiar. Desynchronization over the fronto-central and central electrodes in the alpha band, as found in our study, is linked to the activation of the mirror neuron system, and, therefore, a probable indicator for motor processing [40].

Musicians are trained to be rhythmically precise to create temporally coherent sound sequences. In general, both beat perception and preparation for movement of body parts, such as fingers, have been shown to evoke changes in alpha and beta bands [17,22,41]. Further, functional interaction at alpha and beta frequency is linked to motor control. In our study, musicians had decreased alpha and beta synchrony over the electrode pair FC3 –C3 in Music On relative to Music Off. In addition, several electrode pairs of musicians in fronto-central, central, centro-parietal and parieto-occipital electrodes had decreased synchrony in the beta band in Music On. In self-paced motor tasks, beta oscillation is shown to decrease preceding movements by one or more seconds [42,43]. Fujioka and colleagues [22] proposed that beta band activity in the auditory cortex may help to signal timing cues to facilitate motor preparatory processes for sound synchronization and stated that it is highly likely that oscillatory activities are spread spatially across brain areas and are not limited to the auditory cortical sources.

For a dancer, music is comprehensive and collaborative. Music forms a setting in which dancers produce movements that are coherent with (or intentionally in contrast to) the prevailing sound in terms of rhythm, sentiment, and movement style. When freely listening, a dancer might be more focused on the gist of the music than to the sequence of an individual instrument, melody contour, or rhythmic pattern. Importantly, in this study no participant was familiar with the presented choreography, and thus, no dancer could mentally follow a learned sequence of movements to the music. Therefore, the elevated theta and gamma synchrony over several pairs from prefrontal to parieto-occipital electrodes in dancers during Music On relative to Music Off could reflect the activation of higher-level brain processes [23–25].

In our previous paper [15], we considered the possible processes for increased theta synchrony in dancers compared with musicians and laymen during an audiovisual dance performance. In addition to multisensory [28,44,45], emotional [46,47], or mnemonic processing [48,49], theta synchrony might increase in dancers when listening to music due to the interactive state required from the dancer to create with sound. Müller and colleagues [6] studied cortical synchrony during collaborative musical improvisation and suggested a preponderance of delta and theta frequencies in inter-brain synchronization. They showed that inter-brain coupling generally emerges at lower frequencies, but higher frequencies may sometimes be required to support elaborate coordinative actions. Importantly, inter-brain theta synchrony increased also when a musician was only observing his partner improvising. Dancers are shown to entrain better than laymen with short dance movement sequences of an actor [10]. Therefore, a comparative study with EEG of dancers and musicians in collaborative improvisation of both music and dance might bring insight into the reasons behind the enhanced synchrony over the frequency bands of our study.

Fujioka and colleagues [22] noticed in their study of beat perception in the auditory cortex a peak in gamma synchrony, although with a longer latency, also during beat omission. In contrast, in the beta band a similar peak was absent. Fujioka and colleagues reasoned that the gamma activity may reflect an endogenous process related to musical beat encoding and anticipation of the stimulus timing. The largely exogenous processes related to auditory-motor communication may be the origin of beta synchrony and could explain why the power peak was absent in beat omission in the beta band. Anticipation of the rhythm is crucial for a dancer to move in harmony with music, whereas precise auditory-motor processing is indispensable for a musician to maintain the flow of sounds with his instrument. These different approaches to music could explain our results in dancers and musicians.

In addition to different manners of music listening, differences in the brain structure in dancers and musicians may lead to the distribution of synchrony over the frequency bands. Giacosa and colleagues [12] suggest that dance and music training may produce opposite effects on white matter structure. In dancers, the white matter regions, such as corticospinal tract and corpus callosum, had increased diffusivity and reduced fibre coherence. The authors hypothesized that whole-body dance training would increase the number of crossing fibres, the axon diameters, or the fanning of fibres connecting different brain regions. In contrast, the diffusivity was reduced and the coherence of fibres was increased in musicians in the same regions. The fine-tuned movement training of musicians may result in more focused enhancements in the pathways related to movement production. Karpati and colleagues [11] found similarities in the gray matter structure in dancers and musicians, both groups having increased cortical thickness relative to controls in the superior temporal regions. These regions form an important component of the auditory-motor integration network [7,50]. Therefore, the characteristics necessary in both dance and music, such as sensorimotor integration and production of rhythmic movement, may have a greater association with gray matter, while the differences, such as whole-body versus specific fine-tuned movements and producing music versus reacting to it, may emerge from the white matter structures. These differences in the axon fibres in dancers and musicians may influence to the cell assemblies, the function of which leads to the distinctive results of these two groups in our frequency band analysis.

### Dance effects

When professional dancers watch dance, they pay attention to and evaluate the movement from a different perspective than laymen [51]. In our study, laymen were the only group with systematic changes in synchrony related to the dance movement. In silence, especially when watching a nearly motionless dancer, laymen had increased theta and gamma synchrony. Despite the absence of dance movement, dancers may have interpreted the character according to the storyline. Musicians also are familiar with implicit and atmospheric storytelling through arts and may have focused on the protagonist in that context. On the other hand, laymen may have observed the dancer from a general social perspective considering her intentions, thoughts, and emotions or observed her in the spatial context. Both emotional processing and movement in space are associated with enhanced theta synchrony [23,47,52]. Observation of the vast dance movement with music decreased the alpha and beta synchrony in laymen, referring to the processing of rhythm and movement. Beta power is shown to be reduced with observation and imagination of complex dance movement relative to simple non-dance movements, indicating higher cognitive load [53]. The absence of alpha and beta desynchronization in experts might reflect either more efficient neural processing and faster adaptation to these stimuli or attention being directed to music during the audiovisual stimulus, and therefore, no systematic changes occurring in synchrony with dance.

### Limitations

Due to the novel research paradigm and novel analysis methods applied to continuous EEG data, some limitations need to be discussed. Unfortunately, we did not record the participants familiarity to the composition in any questionnaire. Several participants from each subject group spontaneously reported being familiar to the musical composition. Carmen is a well-known composition, and thus, can be assumed that many of the participants had heard at least parts of it before. Thus, familiarity to the musical composition could play a role, for example, in the results in which only the laymen had statistically significant differences related to dance but dancers and musicians did not, which is somewhat in contradiction with the prevailing results of expertise, see [8] for a review.

Since the participants were not given any specific task or listening method for the stimuli, the groups of participants might have differed in their attentional involvement in the different conditions, based on their background and familiarity with a given stimulation. This bias would be important to control upon in future studies by giving either a cognitive or a preference task to the participants during the sessions as a contrast to free listening.

The EEG sensor-level analyses are seriously confounded by signal-mixing [54]. In general, changes in connectivity between sensors could arise from many different scenarios. These scenarios may be caused by the complex interplay of changes of power and coupling of one or several brain areas, and/or noise. Some of these scenarios may not involve any connectivity change between two brain areas. Changes in the local oscillatory power are accompanied with changes in the signal-to-noise ratio and may lead to increase in detected inter-electrode synchrony even in the absence of any increase in the actual synchrony.

These complications lead to recommendation to perform MEG/EEG connectivity analysis also on the level of source activations to complement the sensor-level analysis [54]. Therefore, in our study, analysis of power changes between conditions, based on ICA components for example, would have complemented the phase synchrony analysis. Then, the results could be interpreted more profoundly by combining the observations of both analyses. However, although source connectivity analysis alleviates the problem of field spread to a certain extent, it does not provide a perfect solution [54]. Therefore, it is crucial to replicate these novel analyses of our study with another EEG data collected with other participants and during different set of dance and music stimuli.

### Conclusions

Our results suggest different processes to take place during free music listening in dancers versus musicians. In addition, music might overcome visual movement during multimodal perception of dance and music among these groups of experts. Laymen, instead, process the movement stimuli in accordance with the results of earlier studies, in which alpha and beta synchrony decreased during movement observation [19,40,53]. Those cognitive and affective processes which underlie increased theta synchrony in laymen when watching a nearly motionless dancer need to be specified in future research. All in all, professional dance and music expertise seems to shape the perception of both music and dance. The EEG analysis method used in our study could be applied in music and dance therapy as well as in educational contexts to understand and utilize brain plasticity under such motorically, cognitively and emotionally demanding task such as dancing and playing music.
